# Department of Public Health

**Published:** 1879-02-20

**Authors:** 


### *A BILL TO ESTABLISH A DEPARTMENT OF PUBLIC HEALTH.*

The preamble to the bill lately enacted by Congress relating to the public health is as follows:

"*Be it enacted by the Senate and House of Representatives of the United States of America in Congress assembled*, That there shall be established at the seat of government of the United States a Department of Health, the general design and duties of which shall be to acquire and diffuse among the people of the United States useful information on subjects connected with the public health; to direct the establishment and management of efficient sanitary and quarantine systems and regulations throughout the several States and Territories of the United States; to supervise the Marine Hospital Service, and to organize and direct a corps of sanitary engineers competent to superintend all public works so far as their construction may affect the public health."

The second section authorizes the appointment by the President of a Director General of Health, as Chief Executive Officer of Health Department, whose duties pertain to the regulation of the Marine Hospital Service, the management of quarantines, the arrangement of census tables with reference to registration and statistics of births, deaths, marriages, procuring information in regard to climatic, meteorological, geological and other conditions affecting the public health.

The salary of this officer is seven thousand five hundred dollars.

The bill provides also for subordinate officers in the Department, consisting of clerk, chemist, engineers, experts, etc.

